# Supplementary material for: Hypofibrinolysis induced by tranexamic acid does not influence inflammation and mortality in a polymicrobial sepsis model
Source: PLoS One. 2019 Dec 31;14(12):e0226871. doi: 10.1371/journal.pone.0226871 (PMC6938370; doi:10.1371/journal.pone.0226871)
Supplement: S1 Fig — (PDF) [file pone.0226871.s001.pdf]

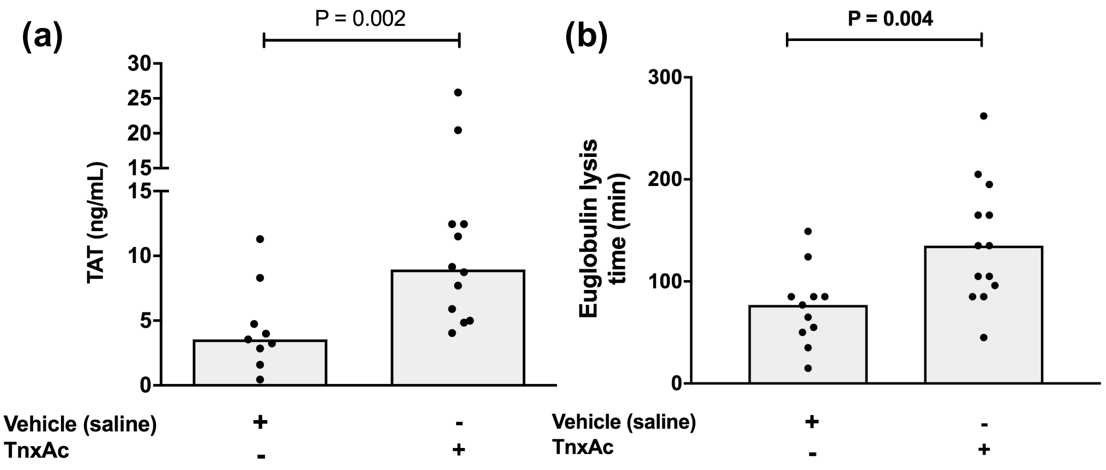


**S1 Fig. Effect of TnxAc on coagulation and fibrinolysis parameters comparing mice treated with vehicle or with TnxAc** (with both doses grouped together)**.** (a) Plasma levels of thrombin-antithrombin (TAT) complexes and (b) the euglobulin lysis time are shown for TnxAc and vehicle treated mice are shown; Mann-Whitney test.
