# Supplementary material for: Hypofibrinolysis induced by tranexamic acid does not influence inflammation and mortality in a polymicrobial sepsis model
Source: PLoS One. 2019 Dec 31;14(12):e0226871. doi: 10.1371/journal.pone.0226871 (PMC6938370; doi:10.1371/journal.pone.0226871)
Supplement: S2 Fig — (PDF) [file pone.0226871.s002.pdf]

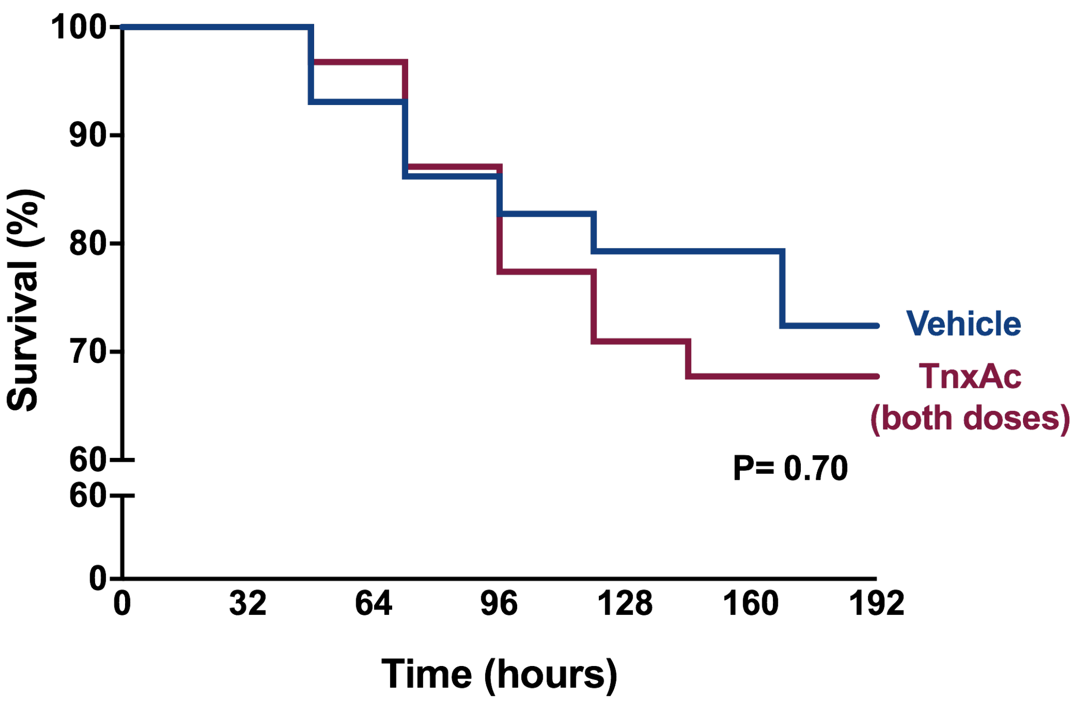

S2 Fig. Survival during polymicrobial sepsis. Kaplan-Meier curves depicting survival of mice treated with TnxAc (both doses grouped together; (n=31) or vehicle (n=29), for up to 7 days; Vehicle (n=29); Log-rank test.
