# Supplementary material for: Hypofibrinolysis induced by tranexamic acid does not influence inflammation and mortality in a polymicrobial sepsis model
Source: PLoS One. 2019 Dec 31;14(12):e0226871. doi: 10.1371/journal.pone.0226871 (PMC6938370; doi:10.1371/journal.pone.0226871)
Supplement: S3 Fig — (PDF) [file pone.0226871.s003.pdf]

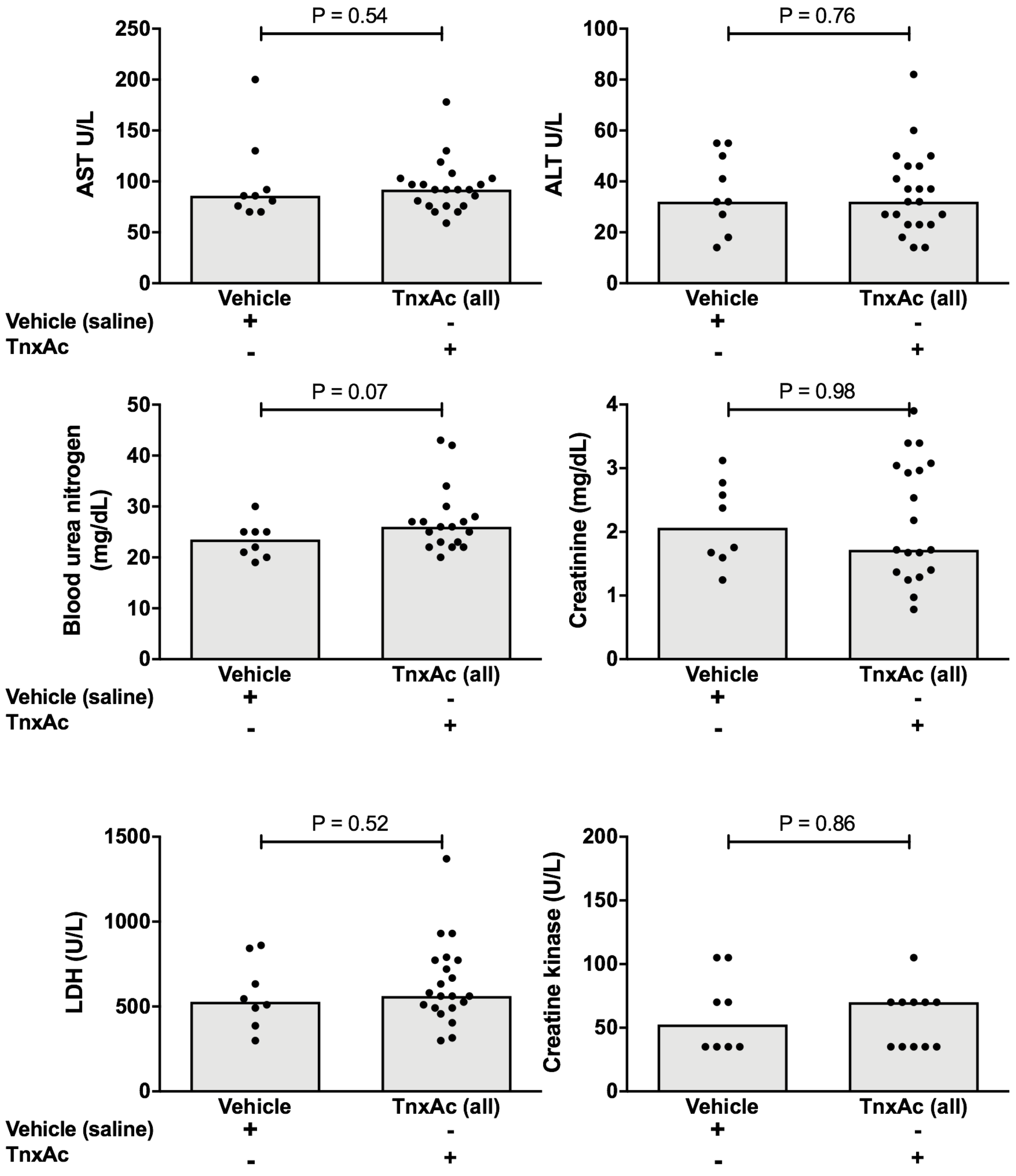

S3 Fig. Biochemical markers of tissue damage comparing mice treated with vehicle or with TnxAc (with both doses grouped together)**.** Median serum levels of (a) AST, (b) ALT, (c) BUN, (d) creatinine, (e) LDH and (f) CK in mice treated with TnxAc or vehicle were measured 24 hours after sepsis induction, and no differences were observed between groups; Mann-Whitney test.
