# Supplementary material for: Hypofibrinolysis induced by tranexamic acid does not influence inflammation and mortality in a polymicrobial sepsis model
Source: PLoS One. 2019 Dec 31;14(12):e0226871. doi: 10.1371/journal.pone.0226871 (PMC6938370; doi:10.1371/journal.pone.0226871)
Supplement: S4 Fig — (PDF) [file pone.0226871.s004.pdf]

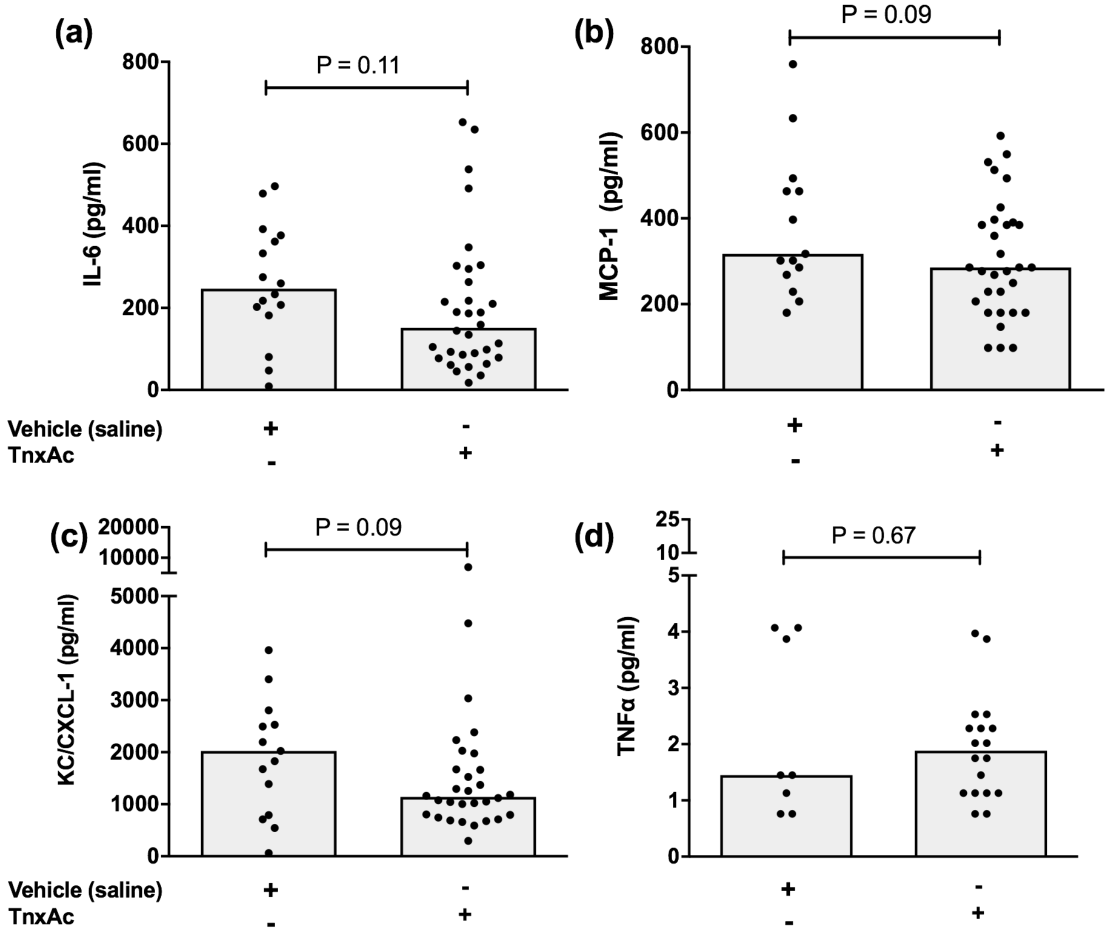

S4 Fig. Inflammatory cytokines and chemokines during polymicrobial sepsis comparing mice treated with vehicle or with TnxAc (with both doses grouped together)**.** Median serum levels of (a) IL-6, (b) MCP-1, (c) KC/CXCL-1 and (d) TNF- α of mice treated with TnxAc or vehicle measured 24 hours after sepsis induction; Mann-Whitney test.
