# Supplementary material for: Hypofibrinolysis induced by tranexamic acid does not influence inflammation and mortality in a polymicrobial sepsis model
Source: PLoS One. 2019 Dec 31;14(12):e0226871. doi: 10.1371/journal.pone.0226871 (PMC6938370; doi:10.1371/journal.pone.0226871)
Supplement: S5 Fig — (PDF) [file pone.0226871.s005.pdf]

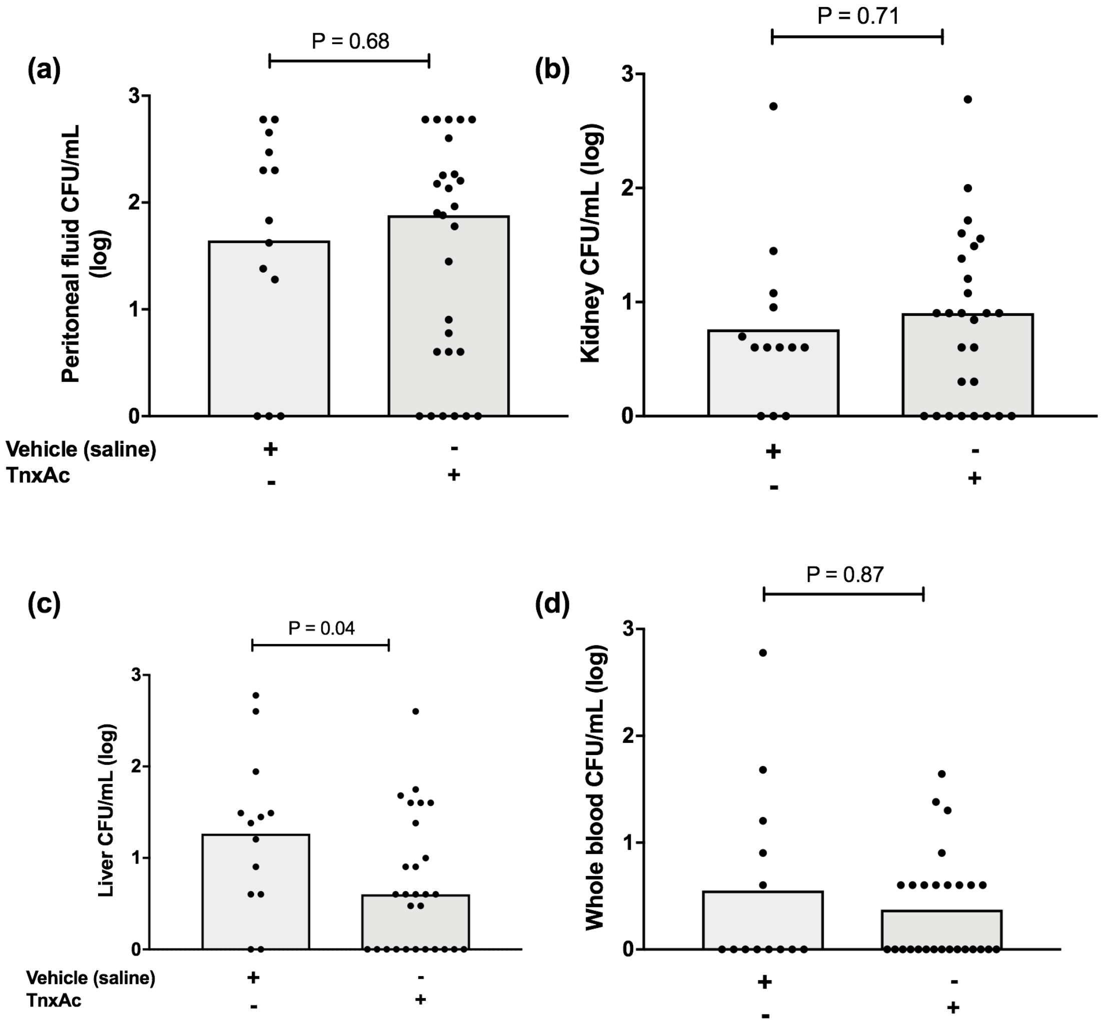

S5 Fig. Bacterial burden in TnxAc treated mice during polymicrobial sepsis comparing mice treated with vehicle or with TnxAc (with both doses grouped together)**.** Mean (log) counts of bacterial colonies per mL in (a) peritoneal fluid, (b) kidneys, (c) liver and (d) whole blood 24 hours after sepsis induction; Mann-Whitney test.
