# Supplementary material for: Hypofibrinolysis induced by tranexamic acid does not influence inflammation and mortality in a polymicrobial sepsis model
Source: PLoS One. 2019 Dec 31;14(12):e0226871. doi: 10.1371/journal.pone.0226871 (PMC6938370; doi:10.1371/journal.pone.0226871)
Supplement: S1 Table — (PDF) [file pone.0226871.s006.pdf]

S1 Table. Effect of TnxAc on hematological parameters (both doses grouped together)

**Parameters**	**Vehicle**	**TnxAc***	****P**
**Hb (g/dL)**	13.1± 1.8	11.6 ± 1.9	0.38
**Platelets ($*10^9$/L)**	902.7 ± 289.1	1033.8 ± 294.7	0.49
**Leukocytes ($*10^9$/L)**	3.47 ± 0.84	4.53 ± 2.36	0.48

* Comparison of vehicle x both groups that were treated with TxnAc (100mg/kg and 600mg/kg doses); **Mann Whitney test.
