# Supplementary material for: Hypofibrinolysis induced by tranexamic acid does not influence inflammation and mortality in a polymicrobial sepsis model
Source: PLoS One. 2019 Dec 31;14(12):e0226871. doi: 10.1371/journal.pone.0226871 (PMC6938370; doi:10.1371/journal.pone.0226871)
Supplement: S2 Table — (PDF) [file pone.0226871.s007.pdf]

S2 Table. Frequency of microvascular thrombosis (both doses grouped together)

Microvascular thrombosis	Vehicle	TnxAc	*P
**Kidneys, n**	4/10	7/19	NS
**Lungs, n (%)**	1/9	0/17	NS
